# Polarity-Dependent Effects of Sinusoidal Galvanic Vestibular Stimulation on Cardiovascular Responses During Cognitive Load

**DOI:** 10.3390/s26175470

**Published:** 2026-08-29

**Authors:** Claudio Zavattaro, Hilary Serra, Emanuele Cirillo, Samuel Cento, Roberto Gammeri, Raffaella Ricci

**Affiliations:** 1 Space, Attention, and Action (SAN) Lab, Department of Psychology, University of Turin, Via Verdi 10, 10124 Turin, Italy; hilary.serra@unito.it (H.S.); emanuele.cirillo@unito.it (E.C.); samuel.cento@unito.it (S.C.); roberto.gammeri@unito.it (R.G.); 2 Department of Physics, University of Trento, Via Sommarive 14, 38123 Trento, Italy

**Keywords:** cardiovascular regulation, galvanic vestibular stimulation, vestibular system, working memory, workload

## Abstract

**Highlights:**

**What are the main findings?**

- Sinusoidal galvanic vestibular stimulation (GVS) induced polarity-dependent autonomic responses during working memory performance.
- Sinusoidal GVS modulated response criterion without changing the sensitivity to targets during performance of a verbal working memory task.

**What are the implications of the main findings?**

- The findings support the hypothesis that vestibular afferents encode sinusoidal GVS in a non-linear fashion, resulting in polarity-dependent effects.
- The findings provide insight into how vestibular afferents interact with cardiovascular regulation under cognitive load.

**Abstract:**

The vestibular system contributes to cardiovascular regulation during changes in gravitational load (e.g., postural transitions, microgravity), in vestibular disorders, and during vestibular stimulations. Studies employing sinusoidal galvanic vestibular stimulation (GVS) reported conflicting autonomic responses, and recent evidence suggests that vestibular afferents encode GVS in a non-linear fashion. Whether this non-linear encoding results in polarity-dependent autonomic responses and whether such responses interact with concurrent cognitive demand remains unknown. To investigate these issues, we used sinusoidal GVS to modulate vestibular input in 35 healthy individuals while physiological signals were recorded using a wearable device. Participants completed a working memory task, preceded and followed by rest periods, under three counterbalanced conditions: right-anodal/left-cathodal GVS (RGVS), left-anodal/right-cathodal GVS (LGVS), and Sham GVS. Perceived stress was repeatedly assessed. During task performance, heart rate increased with RGVS and decreased with LGVS relative to Sham (*p* = 0.001) and perceived stress increased across all conditions (*p* < 0.001). Post-task, heart rate remained elevated in RGVS compared to LGVS (*p* = 0.006), and stress ratings decreased in LGVS compared to the other conditions (*p* < 0.01). These findings indicate that sinusoidal GVS induces polarity-dependent autonomic effects, primarily during concurrent cognitive demand. This pattern may reflect non-linear vestibular encoding, with its emergence being potentially modulated by attentional shifts.

## 1. Introduction

The vestibular system, by encoding head movements (linear and angular acceleration) and gravity, provides feedforward signals that contribute to cardiovascular regulation. During postural transitions from lying to standing, the vestibulosympathetic reflex (VSR) increases the muscle sympathetic nerve activity (MSNA), eliciting vasoconstriction [1,2,3] and maintaining adequate cerebral perfusion [4,5]. In microgravity or ground-based microgravity analogs (e.g., parabolic flights), the unloading of otolith organs reduces the VSR, leading to diminished sympathetic responsiveness and to parasympathetic dominance [6,7,8,9,10]. Thus, the vestibular input plays a critical role in regulating autonomic responses to changes in gravitational load. Beyond gravitational contexts, alterations in the vestibular system per se have been shown to modulate the cardiovascular response. Patients with vestibular disorders present impaired autonomic regulation, characterized by higher sympathetic dominance and related symptoms [11]. In parallel, both animal and human studies report cardiovascular changes associated with experimental modulations of the vestibular system induced by galvanic vestibular stimulation (GVS; [5]).

However, studies investigating the GVS-induced autonomic effects produced conflicting results, particularly concerning heart rate (HR) modulation. Human studies employing binaural bipolar sinusoidal GVS with right-anodal/left-cathodal configuration (RGVS) consistently reported entrainment of sympathetic burst in both MSNA and skin sympathetic nerve activity (SSNA), without observing changes in heart rate (HR) or blood pressure [2,12,13,14,15]. In contrast with these results, other animal and human studies using sinusoidal GVS have found reduced HR and blood pressure during stimulation [16,17]. However, the electrode configuration in those studies was not specified, precluding direct comparison with studies that defined stimulation polarity. Interestingly, similar reductions in HR were observed in a human study employing left-anodal/right-cathodal configuration (LGVS) with a direct current (DC) stimulation [18].

The conflicting findings observed with sinusoidal GVS may reflect differences in sample size, experimental protocols, or electrode characteristics [5]. However, they may also arise from asymmetries in vestibular afferent responses to the anodal and cathodal phases of sinusoidal GVS. Indeed, the conventional view is that vestibular afferent responses to GVS are linear, with the anode reducing afferent signal and the cathode increasing afferent signal [19,20,21,22]. According to this view, equal and opposite physiological responses should be produced by reversing the current flow. However, recent findings in primates demonstrated that vestibular afferents, and particularly irregular fibers [23], encode GVS with marked non-linear patterns, with the increased firing rate induced by cathodal current being significantly higher compared to the decreased firing rate induced by anodal current [22]. Crucially, these non-linear patterns were observed during both DC and sinusoidal stimulations. During sinusoidal GVS, greater response gain was found in the cathodal half-cycle than in the anodal half-cycle for both otolith organs and semicircular canal afferents [22]. Even these subtle asymmetries in vestibular afferent response gain may bias the integration of vestibular signals within vestibulo-autonomic pathways, ultimately generating distinct autonomic responses. Consistent with this possibility, a recent study reported polarity-dependent effects of sinusoidal GVS on motion sickness symptoms within the same individuals [24]. Therefore, reversing stimulation polarity (e.g., RGVS versus LGVS) may not produce physiologically equivalent vestibular activation, potentially explaining the divergent autonomic responses observed in human experimental settings. Thus, directly comparing sinusoidal RGVS and LGVS configurations within the same healthy participants is crucial to clarify whether reversing stimulation polarity differentially modulates cardiovascular responses.

Another relevant yet largely unexplored topic concerns the contribution of the vestibular system to cardiovascular regulation under a concurrent cognitive task. Accumulating evidence indicates that vestibular processing supports cognitive functions. Vestibular alterations have been shown to affect spatial cognition [25,26,27,28,29], tactile perception [30], action and face recognition [31,32], and working memory function [33,34]. Notably, vestibular, cognitive, and autonomic processes share partially overlapping neural substrates, including insular cortices [3,15], suggesting that cognitive load may modulate the vestibular contribution to cardiovascular regulation. For instance, combining head-down rotation (HDR) with mental stress induced by an arithmetic task increases MSNA more than either condition alone [35]. However, how vestibular-induced cardiovascular changes interact with concurrent cognitive demands remains largely unknown.

To address these gaps, we employed binaural bipolar sinusoidal GVS to transiently modulate the vestibular input in 35 healthy individuals. Right-anodal/left-cathodal GVS (RGVS) and left-anodal/right-cathodal GVS (LGVS) protocols were employed to investigate polarity-dependent effects on their cardiovascular response. Autonomic parameters were measured by means of a non-invasive wearable device that reduced participant burden, while providing a reliable estimate of cardiovascular activity. To assess whether cognitive load influences the vestibular-induced cardiovascular response, GVS was applied to participants both at rest and during performance of a cognitively demanding working memory (WM) task. Lastly, to take into account putative emotional shifts and potential stress induced by task performance and/or vestibular modulations, the participants rated their perceived stress at multiple time points and completed an emotional state questionnaire for each GVS condition. At the physiological level, we predicted opposite modulations of autonomic activity during RGVS and LGVS, with the former increasing heart rate and the latter decreasing it. This prediction is based on previous studies reporting increased heart rate during sinusoidal RGVS [14] and decreased heart rate during DC LGVS [18], together with recent evidence demonstrating polarity-dependent effects of sinusoidal GVS on motion sickness symptoms [24]. Finally, we expected the psychological state to mirror physiological arousal [36], with increased stress, high-arousal and negative emotions during RGVS, while increased low-arousal and positive emotions during LGVS.

## 2. Materials and Methods

### 2.1. Experimental Setup

#### 2.1.1. Participants

On the basis of previous GVS studies investigating cardiovascular or cognitive changes [18,31], the sample size was determined to be 25 participants. Thirty-five volunteers were initially recruited to account for potential exclusion of participants. All participants provided informed consent prior to participation and were screened according to specific exclusion criteria. These included the absence of neurological, otological, or psychiatric disorders, and the absence of metallic implants in the body, as well as of pacemakers or other medical devices whose functionality can be altered by GVS. Five of 35 participants (14.28%) were excluded due to missing experimental data (*n* = 3) or lack of vestibular sensations or body movement at 2 mA (*n* = 2), which represented the upper limit of stimulation current chosen to minimize vestibular-related symptoms (e.g., dizziness, nausea, and motion sickness). In the end, the data of 30 participants (21F; mean age 25.57 ± 3.05) were analyzed. The study was approved by the ethical committee of the University of Turin (approval number and date: 0202625 on 3 April 2024) and followed the Declaration of Helsinki.

#### 2.1.2. Physiological Measures

An Empatica E4 wristband (Empatica Srl, Milan, Italy) was used to continuously collect blood volume pulse (BVP) at 64 Hz through the photoplethysmography technique. From the BVP signal, the device used a proprietary algorithm to automatically derive heart rate (HR) and inter-beat intervals (IBIs). Therefore, no additional filters were applied to the signals [37]. However, to ensure the quality of the physiological signals included in the analyses, data completeness was estimated separately for HR and IBIs. Data completeness was calculated as the percentage of observations falling within physiologically plausible ranges. These ranges were set at 40–180 bpm and at 300–2000 ms for IBIs [38]. In all experimental conditions, more than 99% of HR and IBIs observations fell within the specified ranges, indicating high data completeness and physiological plausibility. From IBIs, temporal and spectral heart rate variability (HRV) indices were calculated with the pyhrv Python (version 3.10) module [39]. In particular, the root mean square of successive difference (RMSSD), and the normalized unit high-frequency (%HF) were calculated. HR was considered as a measure of autonomic activity, and RMSSD as a measure of parasympathetic and specifically vagal activity [40], while %HF as a measure of sympathovagal balance represents the relative distribution of spectral power between LF and HF power bands [41,42].

#### 2.1.3. Cognitive Performance

**Figure 1:**
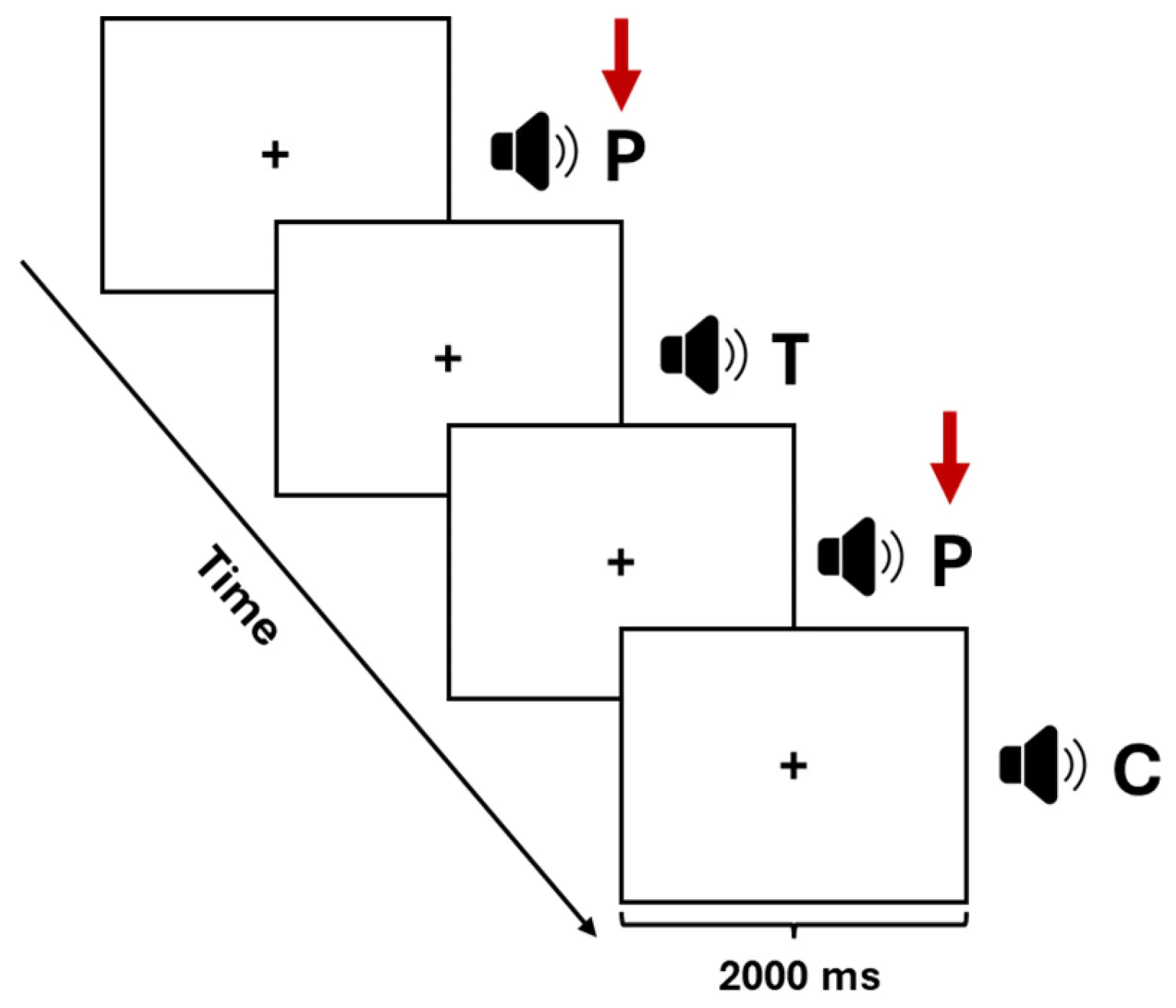
Sequence and timing of events of the verbal 2-back working memory task. Red arrows represent an example of the 2-back rule.

Cognitive load was induced with a verbal working memory (WM) task, and in particular an auditory–verbal 2-back task built with Unity (version 2023.2.14f1). The task consisted of four blocks of 30 trials. In each trial, a letter (C, H, K, M, N, P, Q, T) was randomly presented via auditory modality every 2000 ms (see Figure 1). The participants were instructed to listen carefully to the letters while keeping the gaze on a fixation cross at the center of a computer screen. They had to press the left mouse button only if the heard letter was identical to the one heard 2 trials before. In each block, 4 out of 30 (13.3%) random trials presented a target letter following the 2-back rule. A bip sound determined the end of each block, after which the participants were instructed to press the right mouse button to start the next block (after a brief pause, if necessary). The participants received no feedback about their performance during the whole experimental session. Response times (RTs, the time interval between the letter presentation and the participants’ response), number of Hits (responses to target letters), and number of False Alarms (FA, responses to non-target letters) were analyzed.

In addition, Signal Detection Theory (SDT) analysis was employed to better quantify discriminability index (d’) and response bias (called criterion or c) through Hits and FAs [43,44,45]. Indeed, the n-back task is considered an SDT-based task, in which participants have to detect the presence of a target under uncertainty conditions [46]. In these tasks, the participants’ responses depend on two main variables. The first is their capacity to discriminate the target (signal) from the non-target (noise), which can be quantified with the d’ with the formula.

d′ = Z (Hit rate) − Z (FA rate),(1)

where Z refers to the standardized z-scores. The second is the amount of certainty needed to respond, which can be quantified with the c with the formula.

c = −0.5 × [Z (Hit rate) + Z (FA rate)].(2)

Participants requiring a low certainty to respond adopt a liberal decision-making strategy, with a high number of total responses leading to more Hits, but also to more FAs. Conversely, participants requiring high certainty to respond adopt a conservative decision-making strategy, with a low number of total responses leading to less Hits, but also less FAs. Therefore, the criterion (c) is interpreted as an index of liberal (c < 0), conservative (c > 0) or neutral (c = 0) decision-making strategy [46].

#### 2.1.4. Psychological Assessment

To investigate whether GVS protocols combined with demanding cognitive performance induced changes at the psychological level, perceived stress level, emotional state and subjective mental workload were assessed through questionnaires. Perceived stress level was assessed with a single question (“How much do you feel stressed?”) at different time points during each condition. The participants rated their stress level on a 7-point Likert scale (from “1” = “not at all” to “7” = “extremely”). Emotional state was assessed with a questionnaire based on the Circumplex Model of Affect [47]. The participants rated how they felt in relation to each of 24 emotional adjectives on a 5-point Likert scale (from “1” = “not at all” to “5” = “extremely”). The 24 adjectives were clustered in octants, each representing an emotional valence (positive, negative), an arousal level (high-arousal, low-arousal), or an arousal–valence combination (e.g., low-arousal positive). The median ratings of each emotional octant were analyzed. Perceived mental workload was assessed with the NASA-TLX [48], which is a widely used standardized scale that asks the participants to rate six subscales with a 20 point scale (from “1” = “low” to “20” = “high”). The overall workload score was calculated as the sum of the ratings at the six subscales, in a 20–120 range. Additional questionnaires were included at the beginning of the experimental procedure to take into account participants’ handedness and anxiety traits. Participants’ handedness was assessed with the Edinburgh Handedness Inventory (EHI), in which the participants had to rate whether they typically use the right or left hand to perform 10 daily activities [49]. The handedness score was calculated with the laterality quotient with the formula.

[(R − L)/(R + L) × 100],(3)

where R and L refer to the number of activities performed with the right or left hand, respectively. Participants were considered left-handed in case of laterality quotient below −40, ambidextrous between −40 and +40, and right-handed above +40 [49]. Anxiety trait was assessed with the Beck Anxiety Inventory (BAI), a standardized and validated scale [50]. The participants rated how much they experienced 21 items over the last month on a 4-point scale (from “0” = “not at all” to “3” = “Severely—it bothered me a lot”). The BAI score was calculated as the sum of the ratings at the 21 items, in a 0–63 range. A score of 0–21 was considered as low anxiety, a score of 22–35 as moderate anxiety, and of more than 35 as high anxiety.

To ensure that participants felt the vestibular sensations induced by GVS, at the end of each session, a questionnaire with multiple questions assessed whether they perceived vestibular sensations during the preceding experimental session, whether these sensations were related to illusory movements of the body, and of which type (e.g., translation, rotation) and in which direction (e.g., horizontal, vertical) those illusory movements were perceived.

#### 2.1.5. Galvanic Vestibular Stimulation

Suprathreshold GVS was delivered by means of a stimulator (Soterix Medical, Inc., Woodbridge, NJ, USA) that continuously exerted alternating current with a low-frequency (1 Hz) sinusoidal waveform (amplitude peak ±2 mA) to circular carbon rubber electrodes (1.2 cm diameter). Electrodes were placed over the mastoid processes using plastic holders filled with conductive gel and secured with an elastic strap to prevent movements. The stimulation protocol was binaural and bipolar, and comprised left-anodal/right-cathodal (LGVS), right-anodal/left-cathodal (RGVS), and Sham conditions. In the Sham condition, the current ramped up to the corresponding stimulation intensity in around 10 s to mimic the initial skin sensation of active GVS and then ramped down to 0 mA in around 10 s. The stimulation intensity was determined with a procedure ensuring that each participant received a level of stimulation that was appropriate for their specific sensitivity. During this procedure, the participants were instructed to stand upright, with feet together, eyes closed, and arms relaxed alongside their body. The stimulation amplitude was gradually increased (0.1 mA step) using the stimulator sliding bar, starting from 0.1 mA to maximum 2 mA. The vestibular threshold was considered as the amplitude at which the participants subjectively reported vestibular sensations (e.g., swaying, feeling like on a boat). From this threshold, the amplitude was further increased until the experimenter observed the participants’ bodies swaying. This amplitude was recorded and then the stimulation was gradually decreased to 0 mA. The participants were instructed to reopen their eyes and to move their body before repeating the process two more times, for a total of three times. The stimulation intensity was calculated as the average amplitude recorded in the three trials of the procedure. Stimulation intensity was determined before each GVS condition to account for potential changes in vestibular sensitivity across conditions. Stimulation intensity was highly consistent across conditions (LGVS: 0.947 mA; Sham: 0.974 mA; RGVS: 1.002 mA), indicating only minimal variation in the stimulation amplitude required to elicit the target vestibular response (i.e., the visible body sway).

### 2.2. Experimental Protocol

**Figure 2:**
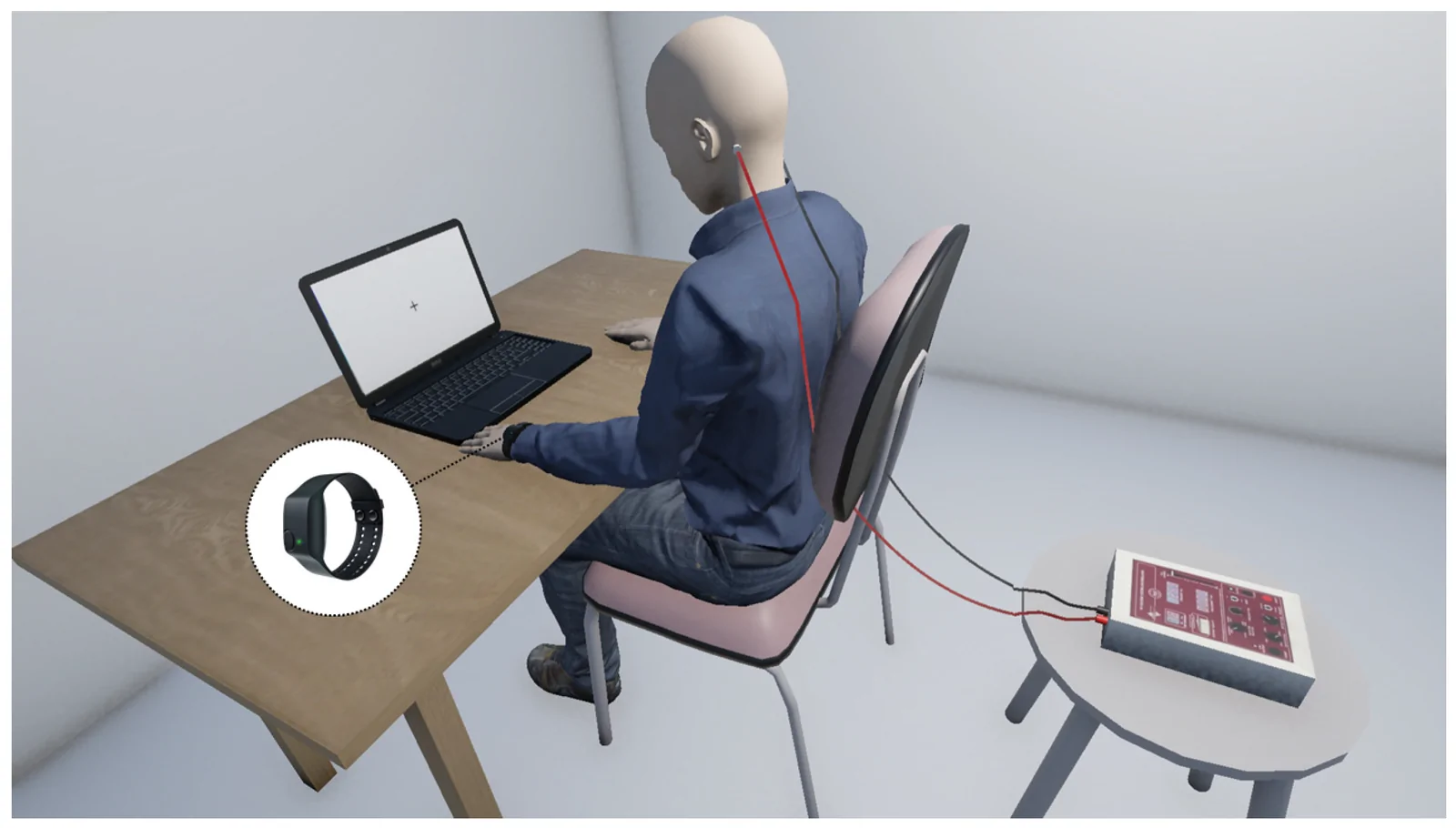
Schematic representation of the experimental setting. The left white circle shows the Empatica E4 wristband. The right part of the figure schematically represents the position of the GVS device during the experimental procedure. The scene was created in Unity (version 6000.0.62f1) using a human avatar generated with MakeHuman (version 1.3.0) for illustrative purposes.

After signing the informed consent and demographic documentation, the participants completed the EHI and BAI questionnaires, and a training of the auditory 2-back task to familiarize with the pace and the difficulty of the task. Then, the participants wore the Empatica E4 wristband on the left wrist and underwent the three GVS conditions (i.e., LGVS, RGVS, and Sham) in an evenly counterbalanced order. In each condition, the vestibular threshold was measured, and then 12–14 min of continuous stimulation was delivered. The participants remained seated while the stimulation was active (see Figure 2).

**Figure 3:**
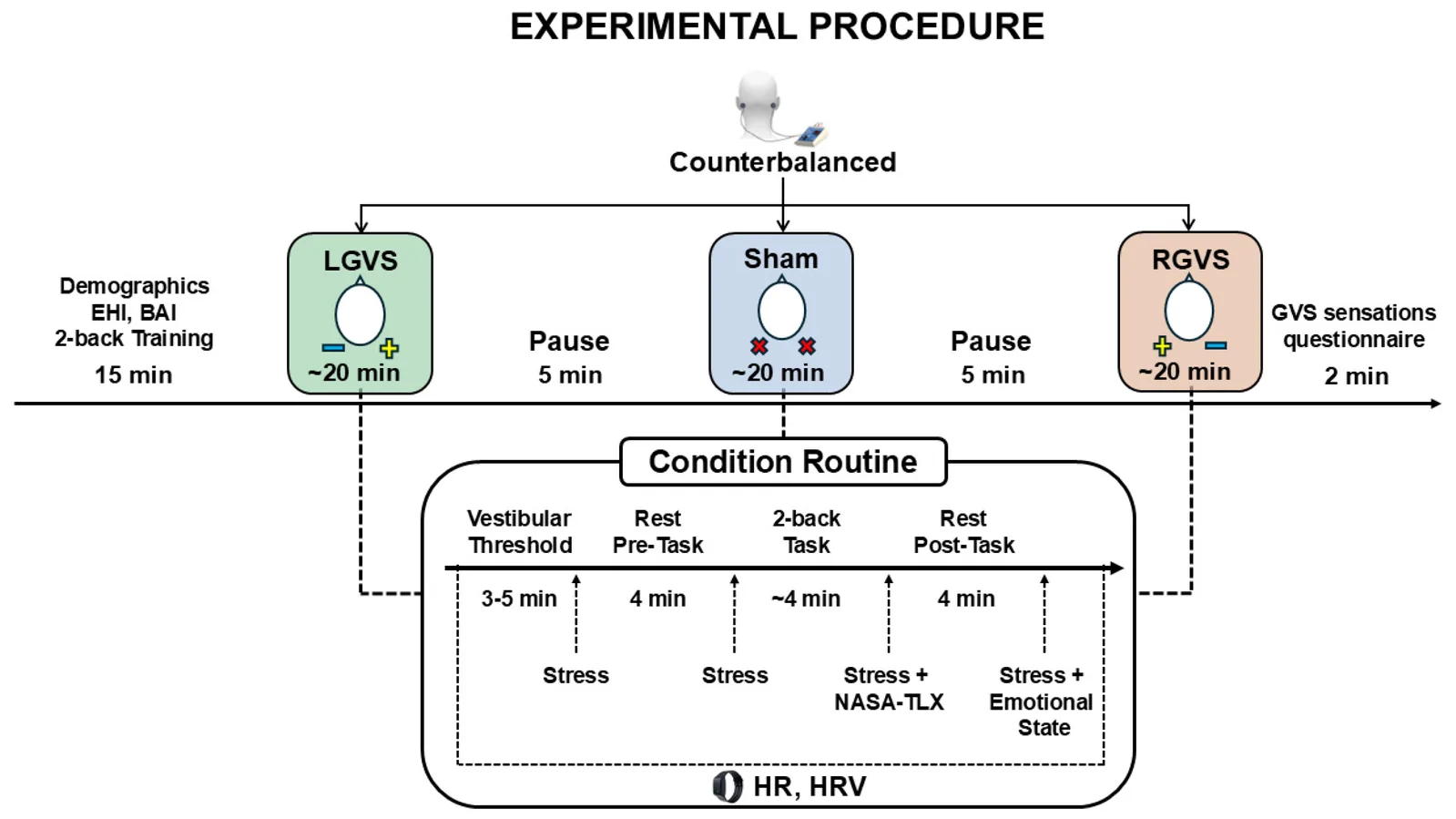
Experimental procedure timeline. BAI = Beck Anxiety Inventory; EHI = Edinburgh Handedness Inventory; GVS = galvanic vestibular stimulation; HR = heart rate; HRV = heart rate variability; LGVS = left-anodal/right-cathodal GVS; RGVS = right-anodal/left-cathodal GVS.

The stimulation time was divided into three periods: a resting period of 4 min before the task (Pre-Task), a period of around 4 min in which they completed the 2-back task (Task), and a resting period of 4 min after the task (Post-Task). At the beginning of the Pre-Task period, and at the end of each period, the participants rated their stress level (4 times per condition). In addition, they completed the NASA-TLX questionnaire after the Task period, and the emotional state questionnaire after the Post-Task period, while stimulated. At the end of each condition, the participants took a 5-min pause. During the whole experimental session, physiological measures were continuously collected. Lastly, they completed the questionnaire assessing the perceived sensations during the stimulation at the end of the experimental session (see Figure 3).

### 2.3. Data Analysis

Data analysis was conducted using SPSS software (version 26). The Shapiro–Wilk test was employed to assess data distribution. In the case of normal distribution, repeated-measures ANOVA (RM-ANOVA) was employed, Friedman tests were conducted otherwise. Post hoc tests were performed using paired t-tests for normally distributed data or Wilcoxon signed-rank tests for non-normally distributed data. A Bonferroni correction was applied in case of pairwise comparisons to reduce the risk of false positive results (type I error). For the cognitive level, RM-ANOVA was used to investigate the main effect of Stimulation (LGVS, Sham, RGVS) on SDT data (d’ and c). On the other hand, the Friedman test was employed on RTs, the number of Hits, and the number of FA. For the physiological level, for HR and RMSSD intra-participant standardization was first applied on HR and RMSSD to reduce the interindividual variability of the data. To this end, z-scores for the three GVS conditions and time periods were calculated for each variable based on the individual mean and standard deviation. This step was not performed on %HF values, as this index is already standardized on a 0–1 scale. Then, for each variable (zHR, zRMSSD, and %HF), the mean value was calculated for each stimulation by aggregating the values across periods, and for each period by aggregating the values across stimulations. At the psychological level, a similar approach was employed for stress level ratings. First, the ratings given at the start of each stimulation condition were subtracted from the ones given at the end of each period. Then, the overall stress rating for each stimulation was calculated as the median delta rating across periods, and the overall stress rating for each period as the median delta rating across stimulations. Three Friedman tests were conducted for all physiological variables (zHR, zRMSSD, and %HF) and stress level ratings in order to investigate the (1) differences between stimulations, (2) differences between periods, and (3) differences between stimulations and periods. For the NASA-TLX and emotional state questionnaires, a Friedman test was conducted on perceived mental workload scores and on the median rating of each emotional octant.

## 3. Results

Regarding handedness, 27 out of 30 (90%) participants were right-handed. Similarly, 27 out of 30 (90%) participants had low anxiety (BAI score < 21), while the remaining 3 had moderate anxiety (BAI score 21–35). Consequently, statistical comparisons between handedness groups and anxiety groups were not conducted. All participants perceived the vestibular sensation induced by GVS, and 28 out of 30 participants (93.33%) reported an illusory movement of the body. This sensation was referred to as bidirectional oscillation (27/28) or as horizontal translation of the body (1/28).

### 3.1. Physiological Results

**Figure 4:**
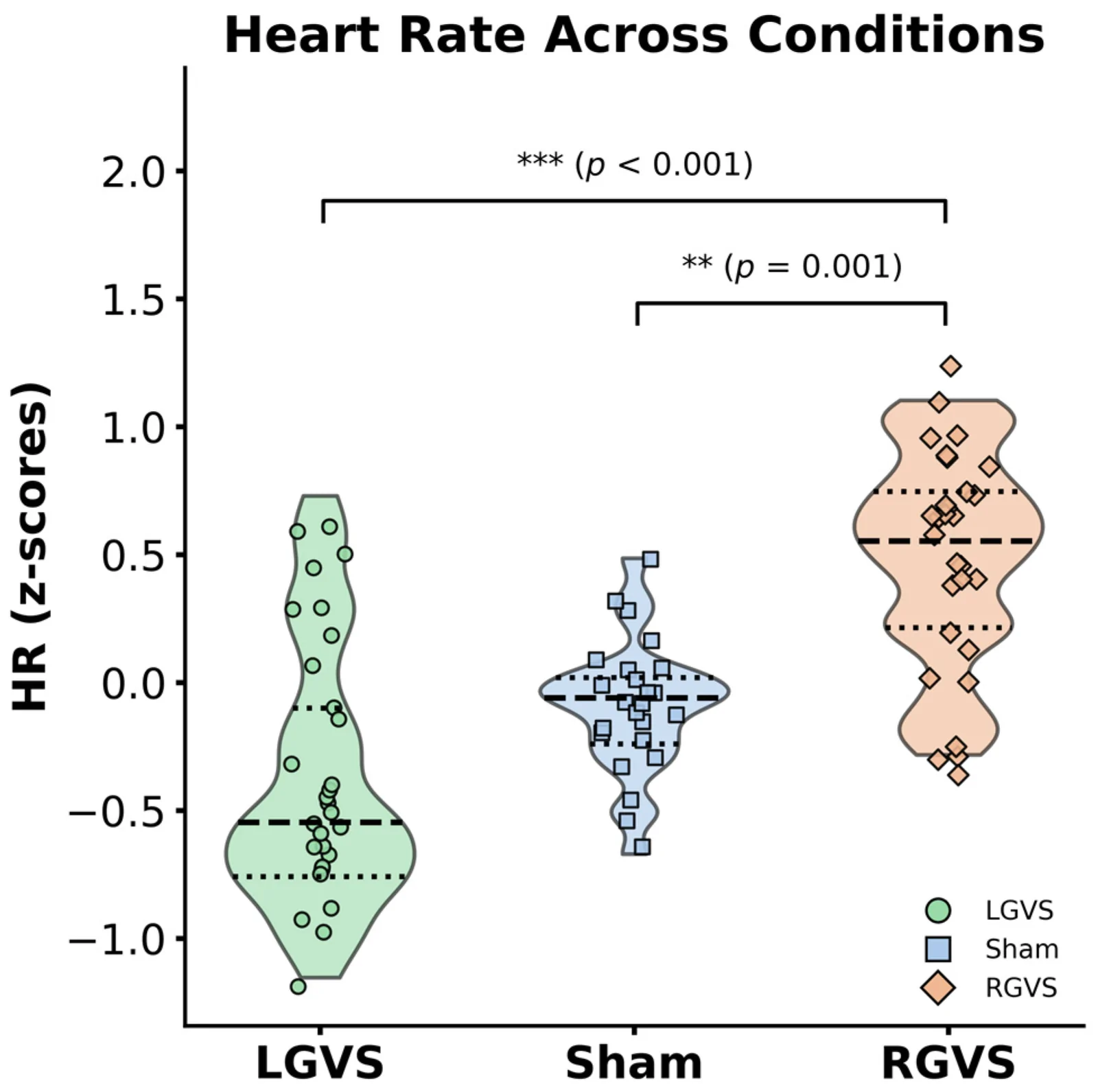
Participants heart rate during each GVS protocol (LGVS, Sham, RGVS). The violin plots represent the data density. The boxplots represent the data distribution with boxes showing the interquartile range with the median represented by the central line, and whiskers indicating the minimum and maximum values. The jitter points represent individual values. ** = *p* < 0.01; *** = *p* < 0.001. GVS = galvanic vestibular stimulation; HR = heart rate; LGVS = left-anodal/right-cathodal GVS; RGVS = right-anodal/left-cathodal GVS.In addition, the Friedman test revealed significant differences between stimulations and periods [χ^2^(8) = 42.809; W = 0.178; *p* < 0.001]. Post hoc tests showed that in the Task period, the participants demonstrated significantly higher zHR during RGVS compared to Sham [z = −3.383; r = 0.618; *p* = 0.001] and LGVS [z = −4.659; r = 0.851; *p* < 0.001], and significantly lower zHR in LGVS compared to Sham [z = −3.301; r = 0.603; *p* = 0.001]. In addition, in the Post-Task period, the participants had a significantly higher zHR during RGVS compared to LGVS [z = −2.725; r = 0.498; *p* = 0.006]. Lastly, only for RGVS participants showed significantly higher zHR during the Task period than during Pre-Task [z = −3.137; r = 0.573; *p* = 0.002] and Post-Task [z = −3.075; r = −561; *p* = 0.002] periods. No significant differences in zHR were observed between periods (see Figure 5A).

The Friedman test revealed significant differences in zHR between stimulations (χ^2^(2) = 14.739; W = 0.246; *p* = 0.001). Post hoc tests showed that participants had significantly higher zHR in RGVS compared to both Sham [z = −3.265; r = 0.596; *p* = 0.001] and LGVS [z = −3.733; r = 0.682; *p* < 0.001] (see Figure 4).

**Figure 5:**
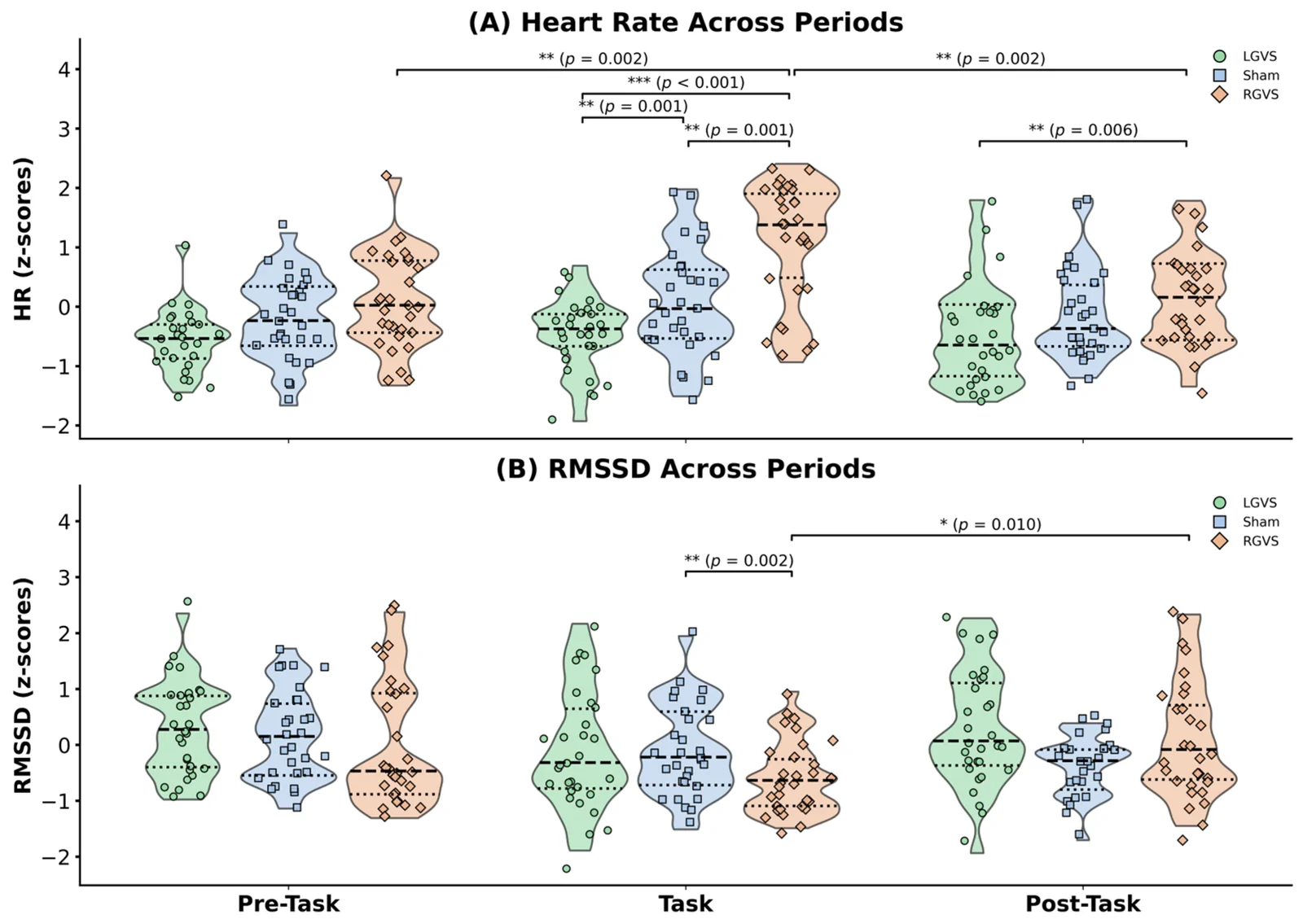
Participants physiological data during each period (Pre-Task, Task, Post-Task) of each GVS protocol (LGVS, Sham, RGVS). (**A**) Heart rate (z-scores) values; (**B**) RMSSD (z-scores) values. The violin plots represent the data density. The boxplots represent the data distribution with boxes showing the interquartile range with the median represented by the central line, and whiskers indicating the minimum and maximum values. The jitter points represent individual values. Significant results are depicted as: * = *p* < 0.05; ** = *p* < 0.01; *** = *p* < 0.001. GVS = galvanic vestibular stimulation; HR = heart rate; LGVS = left-anodal/right-cathodal GVS; RGVS = right-anodal/left-cathodal GVS; RMSSD = root mean square of successive differences.

The Friedman test revealed significant differences in zRMSSD between stimulations and periods [χ^2^(8) = 21.093; W = 0.088; *p* = 0.007]. Post hoc tests showed that during the Task period, participants had a significantly lower zRMSSD during RGVS compared to Sham [z = −3.034; r = 0.554; *p* = 0.002]. In addition, during RGVS, participants had significantly lower zRMMSD in the Task period compared to the Post-Task period [z = −2.561; r = 0.468; *p* = 0.01] (see Figure 5B). No significant differences were observed in %HF with Friedman tests (*p* > 0.05).

### 3.2. Cognitive Results

**Figure 6:**
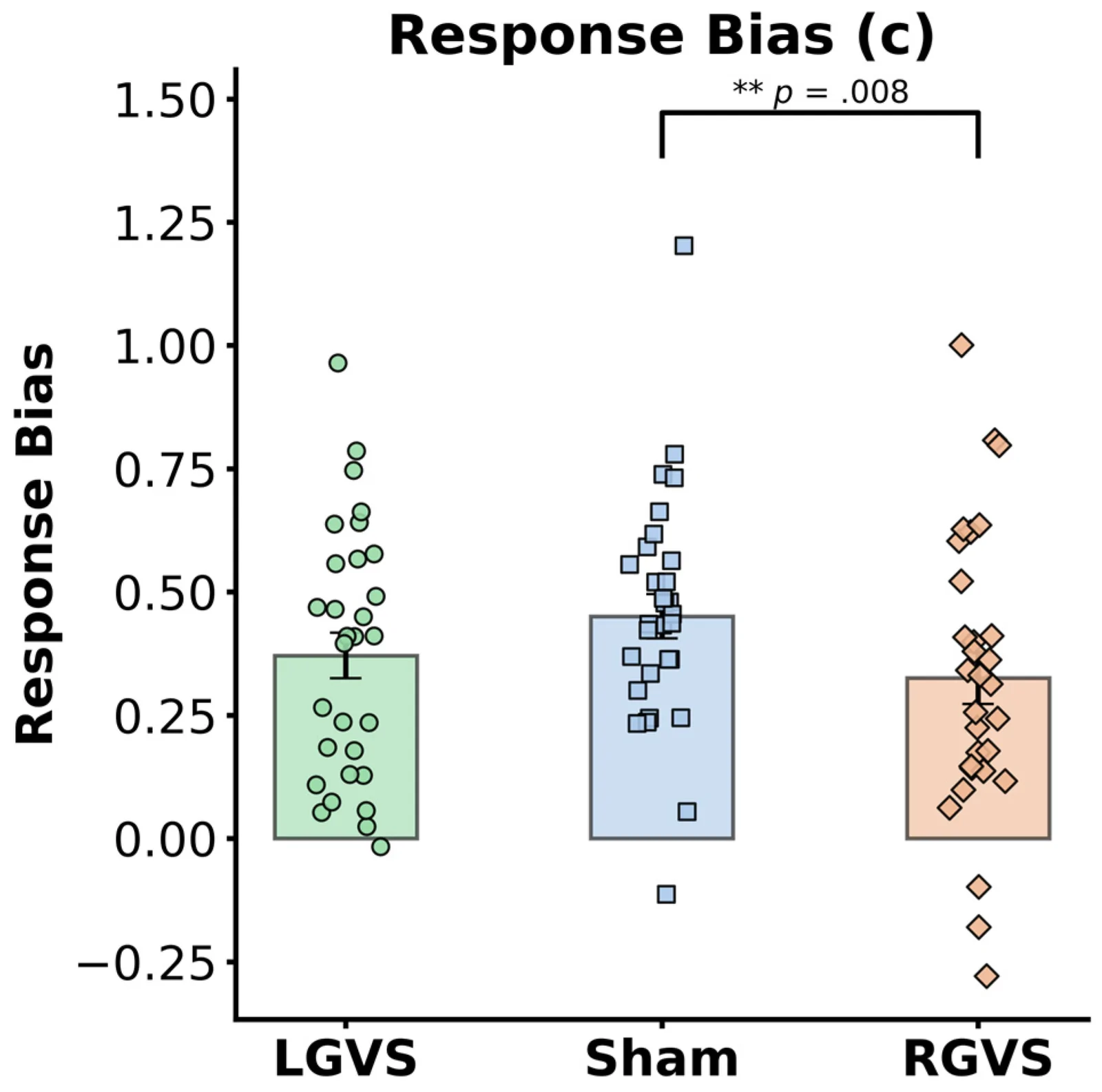
Participants response bias (criterion c) during the performance of the verbal 2-back task in each GVS condition (RGVS, Sham, LGVS). The bars represent the data mean while the whiskers represent the 95% standard error. The jitter points represent individual values. Significant results are depicted as: ** = *p* < 0.01. GVS = galvanic vestibular stimulation; LGVS = left-anodal/right-cathodal GVS; RGVS = right-anodal/left-cathodal GVS.

The Friedman test revealed a significant difference in the number of Hits between Stimulations [χ^2^(2) = 6.608; W = 0.11; *p* = 0.037)]. Post hoc tests showed that the participants had significantly higher Hits during RGVS compared to Sham [z = −1.976; r = 0.361; *p* = 0.048]. However, this result did not survive Bonferroni correction (critical *p* = 0.017). In addition, RM-ANOVA employed on response bias (c) data with Stimulation (LGVS, Sham, RGVS) as main factor and Threshold as covariate revealed a significant main effect of Stimulation [F(2, 27) = 4.507; η^2^_p_ = 0.25; *p* = 0.02]. Post hoc tests showed that c was significantly lower in RGVS compared to Sham [t = −2.83; d = 0.465; *p* = 0.008] (see Figure 6). No significant differences were observed for RTs, FAs, and d’.

### 3.3. Psychological Results

**Figure 7:**
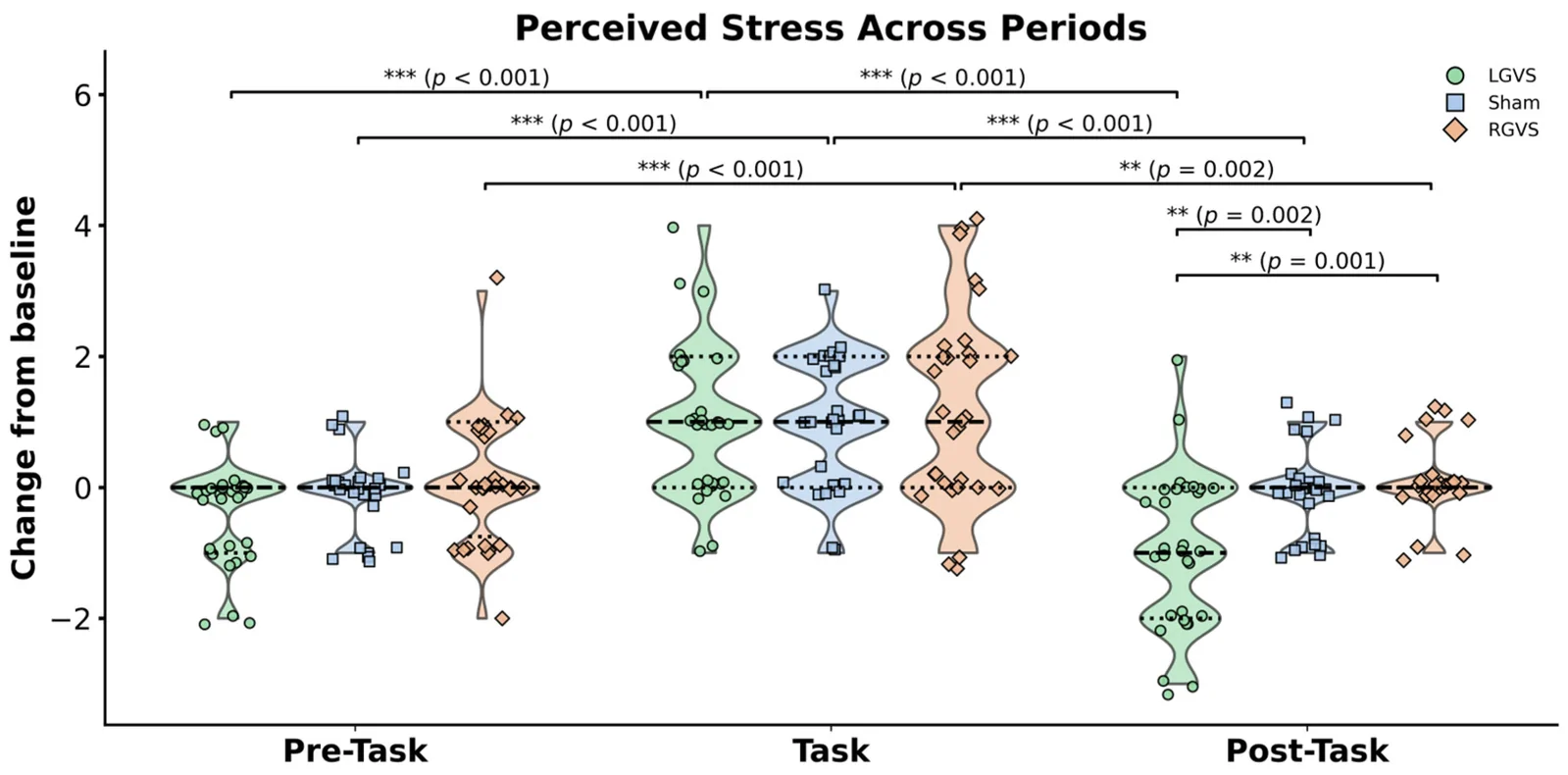
Participants perceived stress level during each period (Pre-Task, Task, Post-Task) of each GVS protocol (LGVS, Sham, RGVS). The violin plots represent the data density. The boxplots represent the data distribution with boxes showing the interquartile range with the median represented by the central line, and whiskers indicating the minimum and maximum values. The jitter points represent individual values. Significant results are depicted as: ** = *p* < 0.01; *** = *p* < 0.001. GVS = galvanic vestibular stimulation; LGVS = left-anodal/right-cathodal GVS; RGVS = right-anodal/left-cathodal GVS.

The Friedman test revealed significant differences in delta stress ratings between periods [χ^2^(2) = 23.922; W = 0.399; *p* < 0.001]. Post hoc tests showed that participants’ delta stress ratings were significantly higher in the Task period compared to both Pre-Task [z = −3.837; r = 0.700; *p* < 0.001] and Post-Task [z = −3.533; r = 0.645; *p* < 0.001] periods. In addition, Friedman test revealed significant differences in delta stress ratings between stimulations and periods [χ^2^(8) = 69.496; W = 0.29; *p* < 0.001]. Post hoc tests showed that during all three stimulations the participants stress ratings were higher in the Task period compared to both Pre-Task [RGVS: z = −3.59; r = 0.655; *p* < 0.001; Sham: z = −4.09; r = 0.747; *p* < 0.001; LGVS: z = −3.579; r = 0.653; *p* < 0.001] and Post-Task [RGVS: z = −3.157; r = 0.576; *p* = 0.002; Sham: z = −3.886; r = 0.709; *p* < 0.001; LGVS: z = −3.669; r = 0.67; *p* < 0.001] periods. In addition, in the Post-Task period, the participants’ stress ratings were significantly lower during LGVS compared to both Sham [z = −3.075; r = 0.561; *p* = 0.002] and RGVS [z = −3.384; r = 0.618; *p* = 0.001] (see Figure 7).

The Friedman test revealed significant differences in emotional state ratings between stimulations only in high-arousal emotions [χ^2^(2) = 6.821; W = 0.114; *p* = 0.033]. Post hoc tests showed that participants had higher high-arousal emotions ratings during RGVS compared to LGVS [z = −2.326; r = 0.425; *p* = 0.02]. However, this result did not survive Bonferroni correction (critical *p* = 0.017). No significant differences were observed in perceived mental workload between stimulations with the Friedman test (*p* > 0.05).

### 3.4. Correlations Between Levels

Spearman correlations revealed significant positive correlations between zHR and perceived stress level (r = 0.185; *p* = 0.002). In addition, significant negative correlations were found between zHR and zRMMSD (r = −0.223; *p* < 0.001), and between perceived stress and zRMMSD (r = −0.177; *p* = 0.003). No correlations were found between response bias and psychophysiological measures in the Task period.

## 4. Discussion

Vestibular system modulations induced by postural transitions, microgravity, or artificial experimental stimulations have been shown to influence cardiovascular responses. Previous studies employing sinusoidal galvanic vestibular stimulation (GVS) produced mixed findings related to cardiovascular modulation. Given the non-linear effects of cathodal and anodal stimulation demonstrated by recent studies [22], it remains unclear whether sinusoidal right-anodal/left-cathodal (RGVS) and left-anodal/right-cathodal (LGVS) GVS protocols may exert differential autonomic effects within the same individuals. Moreover, the vestibular-induced autonomic response under conditions of concurrent cognitive demand remains poorly understood. To address these gaps, this study investigated the effects of suprathreshold sinusoidal GVS on cardiovascular response both at rest and during the performance of a verbal working memory (WM) task. At the cognitive level, the participants shifted their response toward a more liberal criterion in the auditory–verbal 2-back task, with significant results appearing only during RGVS, highlighting the contribution of the vestibular signal in verbal WM. At the physiological level, an overall increase in heart rate was observed during RGVS. Moreover, during cognitive task performance, while RGVS consistently increased heart rate, LGVS decreased heart rate. At the psychological level, higher stress was reported during task performance for both RGVS and LGVS, while after the task the perceived stress level decreased during LGVS, in line with the LGVS physiological finding at rest. No changes in emotional state and perceived workload were observed. Interestingly, perceived stress levels correlated with physiological arousal, while no correlations were found between cognitive performance and psychophysiological state. In the following paragraphs, we discuss the observed results in detail.

### 4.1. Sinusoidal GVS Effects on Verbal Working Memory Task Performance

Vestibular modulation induced by both GVS conditions had no measurable effect on verbal working memory accuracy and response times. However, signal detection analysis revealed that during RGVS the participants employed a more liberal decision-making strategy (reflected by decreased criterion) with no changes in their ability to discriminate (d’) between signal (target letter) and noise (non-target letter). This behavior may reflect greater cognitive demand, as there is evidence that high cognitive load can induce a more liberal response bias without changing the sensitivity to targets [51,52]. However, both RGVS and LGVS showed a comparable trend toward more liberal response criterion, with significant effects being observed only during RGVS. This suggests that the two stimulation polarities may exert effects in verbal working memory that are similar in direction, but different in magnitude. This finding is consistent with recent evidence showing that vestibular afferents encode sinusoidal GVS in a non-linear fashion [22]. Under this framework, the stronger effect elicited by RGVS may reflect a greater engagement of vestibulo-cortical networks involved in verbal working memory, such as the left insular cortex and frontal areas [53,54,55,56]. Neuroimaging studies are needed to confirm this hypothesis.

### 4.2. Polarity-Dependent Cardiovascular Responses During Demanding Cognitive Performance

We predicted a polarity-dependent pattern in autonomic response emerging from RGVS and LGVS. This prediction was partially confirmed. Before the task, no significant autonomic changes were observed for either polarity. During cognitive performance, the predicted pattern emerged, with increased heart rate during RGVS and reduced heart rate during LGVS. After the task, the difference between polarities remained significant, but its magnitude was reduced. These findings indicate that reversing the polarity of sinusoidal GVS generates opposite autonomic responses, primarily during cognitive task engagement. This is again consistent with the assumption that opposite current polarities do not generate equivalent and reversed vestibular responses. Indeed, the non-linear encoding of sinusoidal GVS may lead to differential recruitment of central vestibular networks involved in autonomic control. This possibility is supported by neuroimaging studies employing DC GVS that demonstrated polarity-dependent cortical network recruitment [57,58]. These networks may include several cortical regions that process vestibular signals and contribute to autonomic regulation. For instance the precuneus, a posterior hub of the Default Mode Network (DMN), integrates vestibular information and projects to anterior hubs of DMN that are relevant for autonomic regulation, such as the medial prefrontal cortex (mPFC) [59,60,61,62,63]. Another key example is the insular cortex, which is involved in vestibular processing and contributes to autonomic regulation in a lateralized fashion. Indeed, the right insular cortex is associated with sympathetic modulation [64,65,66,67,68,69], whereas the left insular cortex has been linked to parasympathetic, particularly vagal, regulation [62,64,69,70]. Future studies combining sinusoidal GVS with neuroimaging techniques are required to determine whether the autonomic effects observed in this study are associated with polarity-specific patterns of cortical activation. Moreover, future investigations should also include wearable devices with higher sample rates, to assess minute-by-minute physiological changes induced by GVS, thus better informing on their temporal characteristics.

### 4.3. The Potential Role of the Attentional Focus

The non-linear encoding of sinusoidal GVS may explain the emergence of polarity-dependent effects in autonomic response, but it remains unclear why this pattern primarily emerges during task performance. One possible, albeit speculative, interpretation is that attentional focus may have contributed to the temporal modulation of the autonomic response. During the rest period before the task, participants may have focused more on their internal physiological state, attending to interoceptive signals [71]. Under these conditions, it is possible that the vestibular-induced autonomic changes may have been partially masked by concurrent homeostatic and top-down regulatory mechanisms. Conversely, during task performance, attentional resources were directed outward, toward the task itself, potentially reducing the effect of these regulatory mechanisms and facilitating the emergence of the polarity-dependent autonomic pattern. In the rest period after the task, the pattern persisted with lower magnitude, which may be consistent with a partial reactivation of internal attentional focus and relative regulatory processes, although these mechanisms may not immediately restore baseline physiological responses while the vestibular stimulation is still active. The psychological findings provide some indirect support for this interpretation. Indeed, perceived stress generally mirrored autonomic changes at rest, while it increased across all stimulation conditions during task performance. This pattern may suggest that the factors contributing to perceived stress differed across stimulation periods, with internal physiological sensations potentially contributing more during rest, and task-related cognitive load contributing more during task performance. However, this interpretation remains speculative, as attentional focus and interoceptive processes were not directly measured in this study. Although this interpretation requires direct experimental validation, it may provide a useful framework for generating testable hypotheses about the temporal evolution of the physiological and psychological responses observed in the present study.

### 4.4. Practical Implications

The present findings highlight the importance of considering cardiovascular regulation in the context of vestibular alterations, with potential implications for clinical practice, experimental research, and space medicine. In clinical settings, alterations in cardiovascular regulation should be systematically monitored in patients with vestibular disorders, as cardiovascular alterations may accompany vestibular symptoms and potentially contribute to the overall clinical burden and rehabilitation outcomes. In experimental research, studies employing unilateral or bipolar vestibular stimulations (e.g., galvanic or caloric vestibular stimulations) should account for concurrent cardiovascular changes, as these responses may contribute to cognitive, affective, or neurophysiological alterations. Moreover, studies using sinusoidal GVS should explicitly report stimulation polarity, as RGVS or LGVS may elicit non-linear and asymmetric vestibular responses, potentially contributing to polarity-dependent effects. During spaceflight, microgravity unloads the otolith organs, reducing vestibular afferent signaling, while also inducing cephalad fluid shifts [27,72]. These concurrent alterations in graviceptive input and fluid redistribution may interact to shape the cardiovascular adaptations observed in astronauts. Therefore, investigating the interplay between vestibular processing and cardiovascular regulation during spaceflight is particularly relevant, as their interaction may contribute to the cognitive, psychological and neurophysiological changes experienced by astronauts. Finally, from a sensor perspective, the present findings demonstrate that wrist-worn photoplethysmographic (PPG) devices can provide a reliable and sensitive means of assessing GVS-induced autonomic changes. Compared with conventional ECG-based setups, these wearable devices reduce the burden of electrode placement and wired instrumentation, making them particularly suitable for longitudinal and ecologically valid assessments in clinical and operational settings. In clinical contexts, for example, they could enable the monitoring of cardiovascular responses in vestibular patients during navigation and daily activities, potentially providing insight into autonomic responses associated with postural instability and fall risk. Their unobtrusive nature may also facilitate cardiovascular monitoring in operational environments where conventional instrumentation is less practical, including spaceflight. Overall, these findings highlight the translational potential of commercially available PPG devices for investigating vestibulo-autonomic interactions and monitoring associated cardiovascular responses beyond the laboratory and in real-world conditions.

## 5. Conclusions

The results of this study revealed polarity-dependent effects of sinusoidal GVS on cardiovascular regulation, verbal working memory, and perceived stress levels. This supports the hypothesis that non-linear vestibular afferents encode sinusoidal GVS in a non-linear fashion. Moreover, the presence of a demanding task seems necessary to unveil polarity-dependent effects in autonomic response during sinusoidal stimulation. These findings advance current understanding of the interaction between vestibular processing, autonomic regulation, and cognition, with important implications for clinical populations with vestibular dysfunction, experimental studies employing vestibular stimulations, and space medicine. Further studies are warranted to replicate these findings and elucidate the mechanisms underlying vestibular contribution to cardiovascular regulation during concurrent cognitive task performance.

## Data Availability

The datasets generated and analyzed during the current study are not publicly available due to ethical and privacy considerations but are available from the corresponding author upon request.

## Abbreviations

The following abbreviations are used in this manuscript:

BAI	Beck Anxiety Inventory
BVP	Blood Volume Pulse
DC	Direct current
DMN	Default Mode Network
EHI	Edinburgh Handedness Inventory
FA	False Alarm
HF	High Frequency
HR	Heart Rate
HRV	Heart Rate Variability
GVS	Galvanic Vestibular Stimulation
LGVS	Left-anodal/right-cathodal GVS
mPFC	Medial Prefrontal Cortex
MSNA	Muscle Sympathetic Nerve Activity
RGVS	Right-anodal/left-cathodal GVS
RM-ANOVA	Repeated-measures ANOVA
RT	Response Time
RMSSD	Root Mean Square of Successive Differences
SDT	Signal Detection Theory
SSNA	Skin Sympathetic Nerve Activity
VSR	Vestibulosympathetic Reflex
WM	Working Memory
